# Outcome of monocanalicular intubation for complex congenital nasolacrimal duct obstruction: the role of age

**DOI:** 10.22336/rjo.2022.11

**Published:** 2022

**Authors:** Bahram Eshraghi, Hadi Ghadimi, Safoora Karami, Mojgan Nikdel

**Affiliations:** * Isfahan Eye Research Center, Department of Ophthalmology, Isfahan University of Medical Sciences, Isfahan, Iran; ** Private Ophthalmology Practice, Rasht, Iran

**Keywords:** congenital nasolacrimal duct obstruction, epiphora, probing

## Abstract

**Objective:** Complex congenital nasolacrimal duct obstruction (CNLDO) is caused by pathologies other than the typical incomplete perforation of the thin membrane in the distal end of the nasolacrimal duct (NLD). Our purpose was to determine the success of silicone tube insertion for such complex CNLDO cases.

**Methods:** Children who met the defined criteria for complex CNLDO and underwent monocanalicular silicone tube insertion between April 2016 and December 2020 were included. The tube was retained for 6-8 weeks and the final outcome was measured 3-4 months after tube removal. If the patients were totally symptom free, the outcome was recorded as complete success. Acceptable outcome was defined as Munk score ≤ 1 (requiring less than twice daily dabbing) and others were classified as failed.

**Results:** Initially, 147 eyes of 132 patients underwent NLD intubation. However, after exclusion of the 11 cases with spontaneous tube extrusion (7.48%), 136 eyes of 121 patients entered the final analysis. The mean age was 23.9 ± 13.0 months (range 8-73 months). The outcome was complete success in 100 eyes (73.5%), acceptable in 16 (11.8%), and failure in 20 eyes (14.7%). The differences in the outcome of the procedure for the eyes based on history of previous probing and age was not statistically significant.

**Conclusions:** NLD intubation with monocanalicular stent is effective in the resolution of complex CNLDO in 85% of cases. This procedure is associated with infrequent complications, like tube loss. The success is not negatively affected by older age and previously failed probing history.

**Abbreviations:** CNLDO = congenital nasolacrimal duct obstruction, NLD = nasolacrimal duct

## Introduction

Congenital nasolacrimal duct obstruction (CNLDO) is a common problem, affecting 6% of normal infants, causing symptoms of persistent tearing or eye discharge during the first year of life [**1**,**2**], and frequently associated with risk factors for amblyopia [**3**,**4**]. The rate of spontaneous resolution by 12 months is 90%, and those who persist beyond this age are less likely to resolve without treatment [**5**]. However, many ophthalmologists consider probing for treatment of CNLDO in children as early as 6-10 months [**1**,**2**,**6**,**7**]. Nasolacrimal duct (NLD) intubation is routinely used both for cases with previously failed probing and as a primary treatment for children whose first procedure is performed under general anesthesia, especially in the presence of risk factors for probing failure, like anatomic abnormalities or tight obstructions [**8**]. 

The pathology in CNLDO typically lies in the distal end of NLD, where there is an imperforate membrane at the valve of Hasner [**2**]. In such cases, the obstruction can readily be overcome by minimal pressure exerted by the probe for perforation of the thin membrane. These are known as simple CNLDO, while various other pathologies can arbitrarily be grouped as complex CNLDO [**9**]. In complex (or complicated) CNLDO, various treatment modalities may be used, including NLD intubation, balloon dacryoplasty, endoscopic interventions and dacryocystorhinostomy [**10**]. 

The aim of this study was to determine the success rate of NLD intubation in complex CNLDO and compare our criteria and findings with previous studies. 

## Materials and Methods

This study adhered to the tenets of the World’s Medical Association Declaration, issued in Helsinki and the institutional review board of Isfahan University of Medical Sciences, Isfahan, Iran, granted ethical approval (#IR.MUI.MED.REC.1400.517). Written informed consent was obtained from the parents or legal guardians of the participants. In this retrospective interventional cases series, patients with complex CNLDO, who underwent NLD intubation in a tertiary ophthalmology center or the private oculoplastic clinic of the senior author between April 2016 and December 2020, were included. CNLDO was clinically diagnosed based on the parents’ complaints of persistent tearing and/ or discharge and examination, which proved increased tear lake and matting of eyelashes. Some patients were referred to us with history of failed NLD probing, while others were treated for the first time. Exclusion criteria were the presence of any of the following: tearing due to etiologies other than the lacrimal system involvement (e.g., conjunctivitis, glaucoma, or eyelid abnormalities), lacrimal obstruction proximal to NLD (e.g., punctal agenesis or canalicular obstruction), traumatic NLDO, craniofacial syndromes and complete bony obstruction, preventing passage of any size of probe through the NLD. We defined complex CNLDO as having either a thick membranous obstruction at the valve of Hasner, multiple membranes along the NLD, or bony obstruction of NLD (in a single point or multiple locations along the NLD).

All operations were performed under general anesthesia by a single surgeon and Monoka Fayet silicone tube (Guide of Crawford, FCI ophthalmics, Paris, France) was used for NLD intubation if probing revealed the presence of any of the above-mentioned criteria (subjectively, based on the tactile sense of the surgeon) for complex CNLDO. The olive tip of the metal part was retrieved in the inferior meatus using Crawford hook and after extrusion from the nose, traction of the silicone tube (with relatively high tension) assisted the surgeon to facilitate the insertion of the collarette into the punctum by minimal pushing force (pull-and-push technique), without the need to over-dilate the punctum. 

We did not suture the silicone tube to the lateral nasal wall. Postoperative topical antibiotic and steroid eye drops were prescribed for two weeks. Patients were instructed to return 6-8 weeks after the procedure for re-evaluation and tube removal. Those who did not come back for the follow up were excluded from the study, as well as those whose silicone tubes were missing at the follow up visit (tube loss). The final outcome was determined three to four months after tube removal, taking into account both the subjective (history of persistence or resolution of symptoms of tearing or discharge) and objective data (clinical judgement during the physical examination with particular attention to eyelash matting, discharge and tear meniscus height). If the patients were totally free of symptoms and had normal tear lake, the outcome was recorded as complete success. Acceptable outcome was defined as Munk score ≤ 1 (requiring less than twice daily dabbing) [**11**] and others were classified as failed (needing dabbing more than once a day or worse).

Statistical analysis was performed using SPSS software (IBM SPSS Statistics for Windows, Version 23.0. Armonk, NY). Comparison of outcome between different groups was made using Chi-square or Fisher’s exact tests and p-value of < 0.05 was determined as statistically significant.

## Results

NLD intubation was performed for 147 eyes of 132 patients. However, 11 tubes were spontaneously extruded (7.48%) and these eyes were excluded from the study.

In the final analysis, 136 eyes of 121 patients (67 males (55.4%) and 54 females (44.6%), with 15 cases of bilateral C-NLDO) were included. The mean age was 23.9 ± 13.0 months (range 8-73 months). The patients were categorized into three age groups; 53 were ≤ 18 months (43.8%), 53 were aged 19-35 months (43.8%) and 15 were ≥ 36 months (12.4%). Right and left side involvement were observed in 71 (52.2%) and 65 (47.8%) eyes, respectively.

40 eyes of 37 patients (29.4% and 30.6% of eyes and patients, respectively) presented a history of previously failed probing. The Monoka-Crawford tubes inserted were retained for an average of 63.5 ± 17.1 days (range 46-122 days).

The outcome was complete success in 100 eyes (73.5%), acceptable in 16 (11.8%), and failure in 20 eyes (14.7%). No statistically significant differences were present in the outcome of the procedure for the eyes when compared based on history of previous probing, gender, or age groups (**Table 1**). The only complication observed was mild punctum cheese-wiring (< 2 mm) in two cases.

**Table 1:** Comparison of outcome for different sexes, age groups and history of previous probing

Outcome	Success 100 (73.5%) eyes	Acceptable 16 (11.8%) eyes	Failure 20 (14.7%) eyes	Total 136 eyes (121 patients)	P-value
History of probing					
Positive	31 (77.5%)	3 (7.5%)	6 (15%)	40	0.678
Negative	69 (71.9%)	13 (13.5%)	14 (14.6%)	96	
Age group					
< 18 months	44 (73.3%)	7 (11.7%)	9 (15.0%)	60	0.958
18-36 months	46 (79.3%)	5 (8.6%)	7 (12.1%)	58	
> 36 months	10 (55.6%)	4 (22.2%)	4 (22.2%)	18	
Gender					
Male	47 (70.2%)	9 (13.4%)	11 (16.4%)	67	0.376
Female	43 (79.6%)	4 (7.4%)	7 (13.0%)	54	

## Discussion

CNLDO is often caused by a mucosal block at the Hasner’s valve [**2**]. This type is termed simple CNLDO, while various other pathologies in the NLD are classified as “complex” and account for 16.8-17.8% of all cases of CNLDO [**10**,**12**]. Some of the etiologies include mal-development or non-development of bony NLD (absence or narrowing of bony canal of NLD or blind lacrimal fossa) [**13**], NLD extending lateral to nasal mucosa, thick membranous obstruction of Hasner’s valve, multiple level NLD blocks, diffuse NLD stenosis, fibrosis of NLD, various genetic or craniofacial syndromes (trisomy 21, Crouzon syndrome, Treacher-Collins syndrome, cleft lip, cleft palate, and facial clefts) [**14**,**15**], lateralized or impacted anterior end of inferior turbinate, or buried probe [**10**,**16**]. Various pathologies in the lacrimal system also cause epiphora in the infants and children, but are not anatomically localized to the NLD, so they were not listed among etiologies of complex CNLDO. These include punctal atresia or agenesis, canalicular dysgenesis or stenosis, and atonic lacrimal sac [**10**]. 

Considering the wide range of etiologies grouped under the term of “complex” CNLDO and large differences in the exclusion and inclusion criteria for its definition in literature, different statistics have been reported about the outcome of treatment modalities for complex CNLDO. The surgical approach (conventional vs. endoscopic), follow up duration, and outcome measures have also been different in various reports, further complicating comparison of efficacy of treatment methods. Nevertheless, simple NLD probing is generally agreed to be the least successful intervention, with success rates reported to be 33-36% in complex CNLDO [**9**,**17**]. 

Most surgeons perform NLD intubation, with either bi- or mono-canalicular stents of pulled or pushed types, for complex CNLDO cases. Khatib et al. achieved 71% success rate for treatment of complex CNLDO using pushed monocanalicular stent (Masterka) [**18**]. We have previously reported the success rate in complex NLDO to be 53.3-59.1% for pushed monocanalicular stent (Masterka) [**19**,**20**] and 59.6% vs. 74.4% for pulled monocanalicular (Monoka Fayet) and bicanalicular silicone tubes (Crawford), respectively [**19**]. The success rate with pulled monocanalicular stent in the present study (73.5% complete and 11.8% acceptable) was higher than in our previous study. This could be at least partially explained by the different definitions of complex CNLDO in these studies. In our previous study [**21**], only severe bony obstruction was used to define complex CNLDO, while in the present study, two additional criteria (multiple or thick membranes) were also included. More importantly, our former study had more strict criteria for success (complete resolution of symptoms) assessed at a significantly longer follow up period of 12 months. 

Potential complications related to tube insertion occasionally occur, including corneal abrasion due to stent displacement, granuloma formation and punctal slitting [**22**]. However, in our series, none was observed except for mild punctum cheese-wiring. Another common problem is the early stent dislodgement (tube loss), which is reportedly observed in up to 44-50% of cases [**22**,**23**], but it occurred in only 7.8% of our patients. We believe this is partly due to our method of fixation of punctal plug (pull-and-push technique), unlike other approaches that emphasize on pushing the collarette into the punctum by a second forceps or an inserter.

Another finding of the present study was the similar success rate in different age groups and non-significant relation of success with history of previously failed probing. There is some controversy in literature regarding the prognosis in older children with CNLDO. While some authors have reported decreased success in older children [**18**,**24**-**32**], others have found similar outcomes in children of different ages [**15**,**33**-**35**] and some have emphasized on the role of the type of obstruction (complex vs. simple), rather than the age per se [**36**-**39**]. 

It is of utmost importance to consider the type of obstruction and the treatment modality used for CNLDO in the interpretation of the results of these studies, as most have included a mix patient population without separate reports of success rates for each subgroup. Additionally, some of these articles have used probing, either alone or combined with NLD intubation. On the contrary, to avoid such source of bias, we included only complex CNLDO cases with characteristics associated with a high possibility of response to tube insertion, and this was our single intervention for the patients.

The hypothetic process of self-selection may account for the higher prevalence of complex obstructions among older children, because they do not spontaneously resolve as the child grows [**9**]. Therefore, it is indeed the higher number of complex NLDOs that account for lower success of probing for older children, not the age as an independent factor. Kushner [**9**] first showed that older children can have excellent prognosis if their obstruction is simple, and supported the suggestion of previous authors [**40**], who have related decreased success of treatment of older children to the presence of more complex types of CNLDO among them. 

This hypothesis has been corroborated by further studies from our previous experience [**20**,**21**,**41**], as well as the ones of other authors [**36**-**39**]. Lim et al. included 122 eyes with CNLDO and performed endoscopic-guided bicanalicular silicone tube insertion in the NLD. The increased age of the patient at the time of surgery was not a significant predictive factor for failure of the procedure [**33**]. Kashkouli et al. reported a similar success rate of 33.3% for complex CNLDO treated with probing in both groups of children who were 13-24 months or > 24 months [**17**]. However, the number of complex CNLDO cases in the mentioned study was relatively small, with 15 cases in the two age groups. In another study that included 25 patients with complex CNLDO, the rate of success of probing was 54%, 43% and 48% for children aged 9-12, 12-24, and 24-48 months, respectively [**42**]. 

In contrast to these studies, reports of a less successful outcome of CNLDO treatment for older children are registered. Khatib et al. reported higher success for pushed monocanalicular stent (Masterka) in children with complex CNLDO, who were younger than 24 months (78%), compared to those older than 24 months (57%) [**18**]. This could be partly due to the type of stent used in their study, as pushed stent might bunch up in the lacrimal passages in the presence of complex obstructions of NLD, and is often recommended for simple CNLDO cases [**18**]. Another study that showed poorer prognosis for older CNLDO cases who underwent NLD intubation, was performed on school-age children (7-15 years) that were significantly older compared to our cases [**24**]. Several other studies that have found older age at the time of CNLDO treatment to be a significant prognostic factor for failure to response, have only used probing (without NLD intubation) to overcome the NLD obstruction [**25**-**32**]. Considering the self-selection process and the accumulation of more cases of complex obstructions in older children, probing is insufficient to overcome the stenosis in the lacrimal pathways. Interestingly, some authors have noticed an age-related decline in the success of probing in CNLDO, without such a trend in those who underwent NLD intubation in their series [**34**,**35**].

Our study was limited by its retrospective, non-comparative design. The follow up period was relatively short and the endoscopic approach was not used. Visualization through nasal endoscope would have provided valuable information regarding the abnormalities accounting for complex CNLDO. The comparative design of the study for determining the differences in the outcome of NLD intubation for patients with simple and complex CNLDO in different age groups could better illustrate the contribution of age and type of obstruction on the success of the procedure.

In conclusion, we found NLD intubation with monocanalicular pulled stent (Monoka Fayet, Crawford design) to be effective in the resolution of complex CNLDO in 85% of cases. This procedure was well-tolerated and associated with infrequent complications, like tube loss or punctum cheese-wiring. The success was not affected by older age and previously failed probing history. Larger controlled studies with endoscopic guidance could better illustrate the diverse etiologies for complex CNLDO.

**Conflict of Interest statement**

Authors state no conflict of interest.

**Informed Consent and Human and Animal Rights statement**

Informed consent has been obtained from all individuals included in this study.

**Authorization for the use of human subjects**

Ethical approval: The research related to human use complies with all the relevant national regulations, institutional policies, is in accordance with the tenets of the Helsinki Declaration, and has been approved by the institutional review board of Isfahan University of Medical Sciences, Isfahan, Iran.

**Acknowledgements**

None.

**Sources of funding**

None.

**Disclosures**

None.
